# Cross-disease immune cells atlas reveals the similarities and differences of cell characteristics and interactions in rheumatic diseases

**DOI:** 10.3389/fmed.2026.1820336

**Published:** 2026-05-13

**Authors:** Liqing Ding, Qiming Meng, Ding Bao, Bingying Dai

**Affiliations:** 1 School of Computer Science and Engineering, Central South University, Changsha, Hunan, China; 2 Department of Rheumatology and Immunology, Xiangya Hospital, Central South University, Changsha, Hunan, China; 3 Provincial Clinical Research Center for Rheumatic and Immunologic Diseases, Xiangya Hospital, Changsha, Hunan, China; 4 National Clinical Research Center for Geriatric Disorders, Xiangya Hospital, Changsha, Hunan, China

**Keywords:** cell interactions, cross-disease, immune cells, rheumatic diseases, scRNA-seq

## Abstract

**Introduction:**

Rheumatic diseases are a group of immune-mediated inflammatory disorders, in which abnormal activation of immune cells is closely related to the occurrence and development of the diseases. Previous studies have mostly been limited to single diseases or single cell types, lacking systematic comparisons of immune profiles across multiple diseases and interactions between cell types, which has restricted our understanding of the overall pattern of immune dysregulation in rheumatic diseases.

**Methods:**

To provide a systematic characterization of the similarities and differences in immune cells among various rheumatic diseases, this study integrated single-cell transcriptome data from peripheral blood mononuclear cells of five rheumatic diseases and healthy controls.

**Results:**

Different rheumatic diseases share some immune dysregulation modules, such as impaired function of γδ T cells. We found that the functions of γδ T cells, including immune regulation and cell proliferation, were significantly decreased in five rheumatic diseases. The analysis of cell communication suggested that the MIF and GALECTIN signaling pathways play a key role in the interaction between T cells and myeloid cells in five rheumatic diseases. However, rheumatic diseases retain their specific disease characteristics, such as different myeloid activation states. Among them, the myeloid cells in Behçet’s disease patients were generally found characterized by enhanced cytotoxicity, while they showed active inflammatory states in systemic lupus erythematosus.

**Discussion:**

This study systematically characterized the immune cell characteristics and interaction networks across rheumatic diseases, revealing shared and specific immune dysregulation patterns, and provided new insights into understanding the immune mechanisms of rheumatic diseases and developing targeted therapeutic strategies.

## 1 Introduction

Rheumatic diseases are a heterogeneous group of disorders characterized by abnormal activation of the immune system, chronic inflammation, and multi-organ involvement, including rheumatoid arthritis (RA), systemic lupus erythematosus (SLE), primary Sjögren’s syndrome (pSS), Behçet’s disease (BD), and IgG4-related disease (IgG4-RD) (1). Although these diseases differ in clinical manifestations, affected organs, and immunopathological features, they all involve dysregulation of innate and adaptive immunity, particularly abnormal activation and functional disorders of T cells, B cells, and myeloid cells (2–4). Recent studies suggest that different rheumatic diseases may share some characteristics and regulatory pathways of immune cell subsets, but there are also disease-specific patterns of immune dysregulation, which provide important pathological clues for understanding their commonalities and individualities (5, 6). However, previous studies have mostly been limited to single diseases or single cell types, lacking systematic comparisons of immune profiles across multiple diseases and interactions between cell types, which has restricted our understanding of the overall pattern of immune dysregulation in rheumatic diseases.

The advent of single-cell RNA sequencing (scRNA-seq) technology has provided a revolutionary tool. This tool enables researchers to decipher transcriptomic features, functional states, and intercellular interactions of immune cells at single-cell resolution (7). This technology has been widely applied in the study of immune cell heterogeneity in various rheumatic diseases, revealing disease-specific subpopulations, aberrant activation pathways, and potential therapeutic targets (8–10). However, most studies have focused on individual or two types of diseases, lacking cross-disease integrative analyses that cover a variety of diseases, making it difficult to systematically uncover the commonalities and specific alterations of immune cells among different rheumatic diseases (6, 11–15). Therefore, constructing a single-cell immune cell atlas spanning multiple rheumatic diseases and systematically comparing their cellular composition, functional states, gene expression, and intercellular communication similarities and differences is of significant scientific importance for deepening the understanding of the common immune mechanisms of rheumatic diseases and identifying broad-spectrum or disease-specific immune therapeutic targets.

This study aims to integrate single-cell RNA sequencing (scRNA-seq) data of peripheral blood mononuclear cells (PBMCs) from five rheumatic diseases (BD, IgG4-RD, pSS, RA, SLE) and healthy controls (HC) to construct a cross-disease immune cell atlas, systematically revealing: (1) the changes in the composition and proportion of immune cells among different diseases; (2) the commonalities and specificities of T cells and myeloid cells in terms of functional states and differentially expressed genes; (3) the interaction patterns of signaling pathways among key inflammatory cell types. Through this cross-disease comparative study, we expect to provide new insights into the immunopathological mechanisms of rheumatic diseases and offer data support for future precision immune intervention strategies.

## 2 Materials and methods

### 2.1 Single-cell RNA-seq data collection, preprocessing of PBMCs

Single-cell transcriptomics datasets containing PBMCs, with processed data or processed CellRanger files available, were collected from public repositories (Supplementary Table 1). A total of six public scRNA-seq datasets representing multiple tissues and rheumatic diseases were included in this data collection. The scRNA-seq data we generated were aligned and quantified using the Cell Ranger Single-Cell toolkit (v.9.0.0) against the GRCh38 human reference genome. Preliminary filtered data generated from Cell Ranger were used for downstream analysis. Further quality control was applied to filter out low-quality cells with either (1) <400 or >5,000 expressed genes (2) >15,000 or <1,000 UMIs (3) >10% mitochondrial gene counts (4) <0.8 log10 genes per UMI. The resulting gene expression matrix, once filtered, underwent normalization using Seurat NormalizeData function, with default parameters. Subsequently, we identified the top 2,000 highly variable genes (HVGs) for reciprocal principal component analysis (RPCA). We used RPCA to mitigate batch effects from different sequencing datasets, but the inherent heterogeneity in sample processing, sequencing depth, and donor demographics across the six public datasets may still introduce errors. Selection of Principal Components (PCs) was based on criteria established through elbow and Jackstraw plots. Using the FindClusters function in Seurat R package, we identified clusters, allowing for a range of resolutions between 0.1 and 1, and used the Uniform Manifold Approximation and Projection (UMAP) for visualization. Differential gene expression analysis was conducted across clusters generated at various resolutions through the Wilcoxon rank sum test, using the FindMarkers function.

### 2.2 Data integration and cell subsets annotation

A specific resolution was determined when major cell types emerged as clusters at that particular resolution but not at lower resolutions. The annotation of resulting clusters into major cell types relied on the expression patterns of canonical marker genes, including T cells (CD3D, CD3E), NK cells (KLRF1, NKG7), B cells (CD79A, MS4A1), myeloid cells (LYZ, CD14, CD16, CD68). Sub-clusters of CD4 and CD8 T cells were annotated based on the different markers of T cell types, and pDCs, cDCs, monocytes, and macrophages were annotated based on the different markers of cell types in myeloid cells.

### 2.3 Calculation of gene signature scores

Signatures for T cell status-related gene sets were derived from the built-in reference gene sets of the TCellSI package. And signatures for gene sets related to immune regulation of myeloid cells were derived from previous studies (Supplementary Table 2). To compute gene signature scores on the basis of the scRNA-seq data, individual cells were scored using the GSVA package, which calculated the average expression levels of selected genes at the celltype level.

### 2.4 Distribution of cell clusters across tissues

To assess the distribution of cell clusters across various diseases, we calculated the proportion of each cell cluster among all PBMCs in samples. In addition, we extended our analysis by computing the difference in quantity through the Wilcoxon rank sum test for each cell cluster across diverse diseases.

### 2.5 Gene set enrichment analysis and intercellular communication

Gene set enrichment analyses for T cell clusters were performed by enricher function from clusterProfiler R package with parameter “qvalueCutoff = 0.05.”

CellChat was used to analyze the intercellular communication networks from scRNA-seq s data. To infer the specific ligand–receptor pairs, the most prevalent signaling pathway for further visualization.

## 3 Results

### 3.1 Construction of immune cell scRNA-seq atlas across diverse rheumatic diseases

**FIGURE 1:**
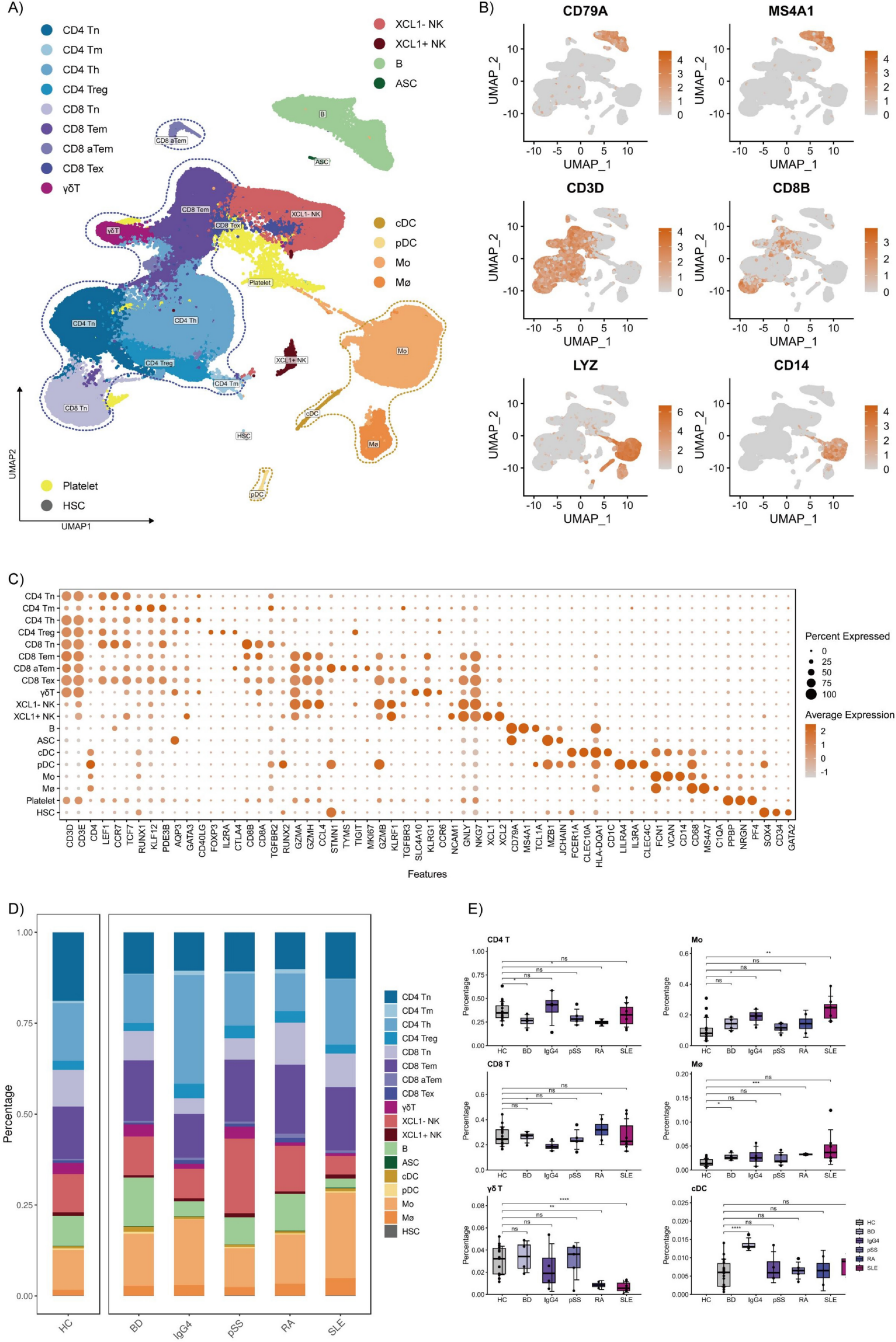
Single-cell RNA sequencing (scRNA-seq) atlas of peripheral blood mononuclear cells (PBMCs) across diverse rheumatic diseases. **(A)** Uniform manifold approximation and projection (UMAP) visualization of PBMCs from all samples. Specific groups are highlighted by dashed circles. **(B)** UMAP plots showing the canonical cell markers. **(C)** Dot plot showing the expression of selected canonical cell markers in annotated cell types. **(D)** Average proportion of each cell type derived from HC, BD, IgG4-RD (IgG4), pSS, RA, and SLE. **(E)** Boxplots comparing the proportion of specific cell clusters in different samples (indicated by colors). *p < 0.05, **p < 0.01, ***p < 0.001, ****p < 0.0001, Wilcoxon test.

To systematically characterize the immune cell heterogeneity in multiple rheumatic diseases, we collected five PBMCs scRNA-seq datasets from previous studies (Figure 1A). Following the quality control for each dataset, we obtained a total of 246,726 single-cell transcriptomes (Supplementary Table 1). This scRNA-seq atlas comprises 40 samples spanning five different diseases. Integrative clustering across all these datasets using reciprocal PCA identified lymphocytes (T cells, natural killer cells, B cells, antibody secreting cells), myeloid cells (pDCs, cDCs, monocytes/Mo, macrophages/Mø), hematopoietic stem cells (HSC) and platelet. According to their canonical gene markers, T cells were categorized into αβ T and γδ T cells, and including CD4 T cells (Tn, Tm,Th, Treg) and CD8 T cells (Tn, Tem, aTem, Tex) (Figures 1B, C). Each sample contained almost all of these immune cell types in various proportions (Supplementary Figures 1A, B). However, we observed composition differences in lymphocytes and myeloid cells between HC and diseases (Figures 1D, E). The proportion of CD4 T cells significantly decreased in BD and RA than in HC, while CD8 T cells significantly decreased in IgG4-RD. Compared with HC and other rheumatic diseases, the proportion of γδ T cells decreased significantly in RA and SLE. As for myeloid cells, macrophages significantly increased in BD and RA compared with HC and other rheumatic diseases. The proportion of monocytes and cDCs also increased in BD.

### 3.2 Similar T cell dysfunction, especially involving γδ T cells, is observed across rheumatic diseases

**FIGURE 2:**
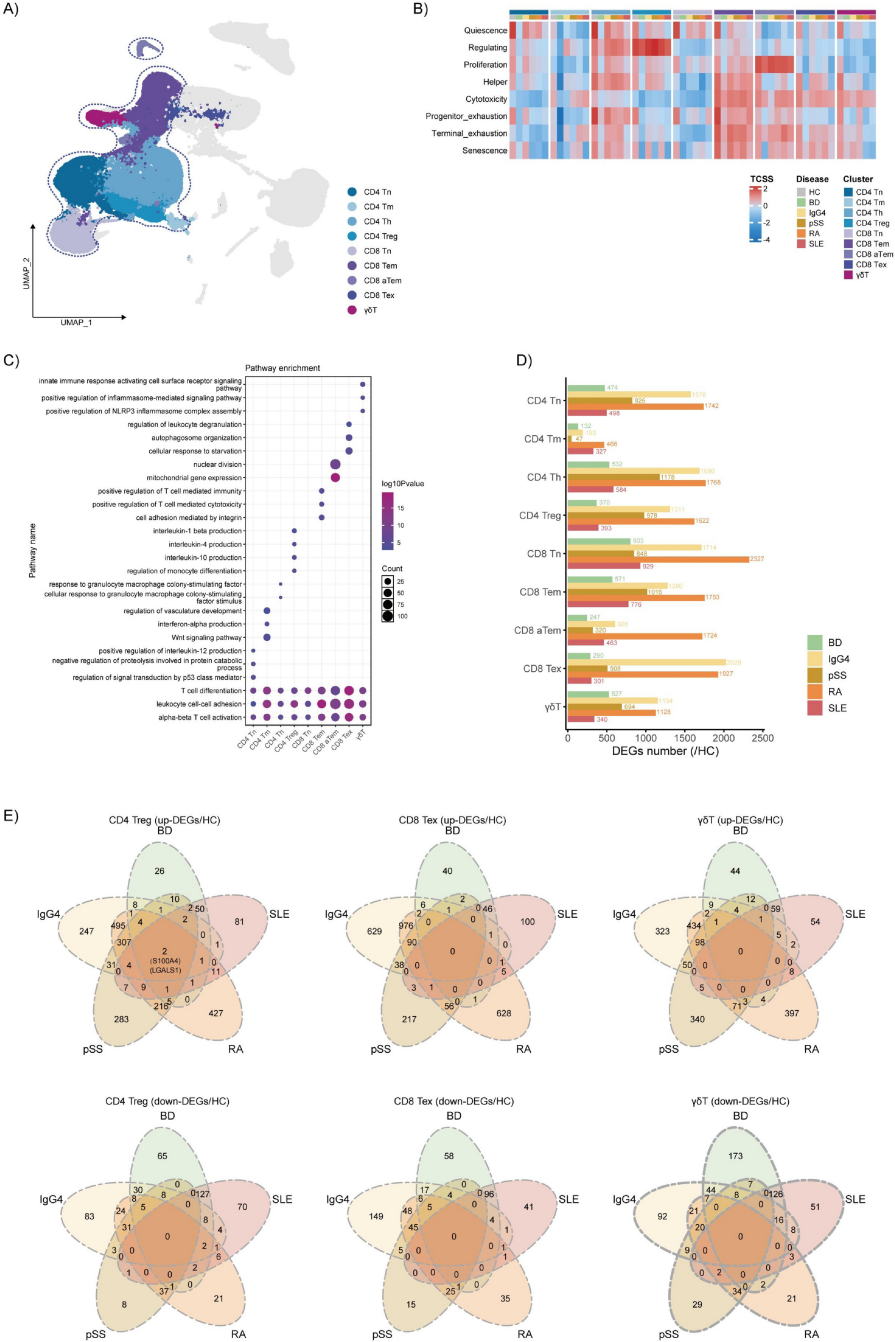
Similar T cell dysfunction in rheumatic diseases. **(A)** Uniform manifold approximation and projection (UMAP) visualization of T cells from all samples. T cell sub-clusters are highlighted by dashed circles. **(B)** Heatmap of T cell status score (TCSS) in each sample and sub-clusters. Scale the data in different TCSS of all sub-clusters. **(C)** GO functional enrichment of each T cell sub-clusters. **(D)** Barplots showing the number of disease-specific differential genes expressions (DEGs) for each T cell sub-cluster among the five diseases. **(E)** Venn plots showing the number of the same and the different disease-specific DEGs in CD4 Treg, CD8 tex and γδ T cells of rheumatic diseases.

To test the hypothesis of shared T cell dysfunction, we next investigated in detail the changes in T cells across the rheumatic diseases (Figure 2A). Based on the labeled T cell status markers in the TCellSI tool, the eight different states of T cell sub-clusters were scored to investigate the similarities and differences in various diseases (Figure 2B and Supplementary Figure 2). In most T cell sub-clusters, cells in HC exhibited higher resting state scores, while each T cell subset in BD had a lower score for each T cell state than HC. Compared with other subsets, Treg has a higher regulatory score. Among different samples, pSS has the highest score for the regulatory state. Each cell state score of Tex and γδ T cells is higher in the cell population of HC. Compared with other cell sub-clusters, CD4 Treg cells are associated with the production of various interleukins (IL-1, IL-4, IL-10), while γδ T cells are involved in innate immunity mediated by NLRP3 (Figure 2C). In order to explore the differential genes expressions (DEGs) of T cell sub-clusters across rheumatic diseases, we compared the disease-specific DEGs for each T cell sub-cluster. Among the five diseases, Ig-G4 RDs and RA has a far greater number of disease-specific DEGs than in any of the other diseases in almost all sub-clusters (Figure 2D). Finally, we focused on the similarities between the up-regulated or down-regulated disease-specific DEGs across different diseases in each T cell sub-clusters (Figure 2E and Supplementary Figure 2). We found that although there were relatively few or even no disease-specific DEGs present simultaneously in all diseases at cell-cluster level, the disease-specific DEGs showed certain similarities in IgG4-RD and RA. LGALS1 and S100A4 were up-regulated in Treg cells of all diseases, while some heat shock proteins, such as HSPD1 were found in the down-regulated of CD8 Tn and Tem in diseases.

### 3.3 Differences in the immune regulation functions of myeloid cells across rheumatic diseases

**FIGURE 3:**
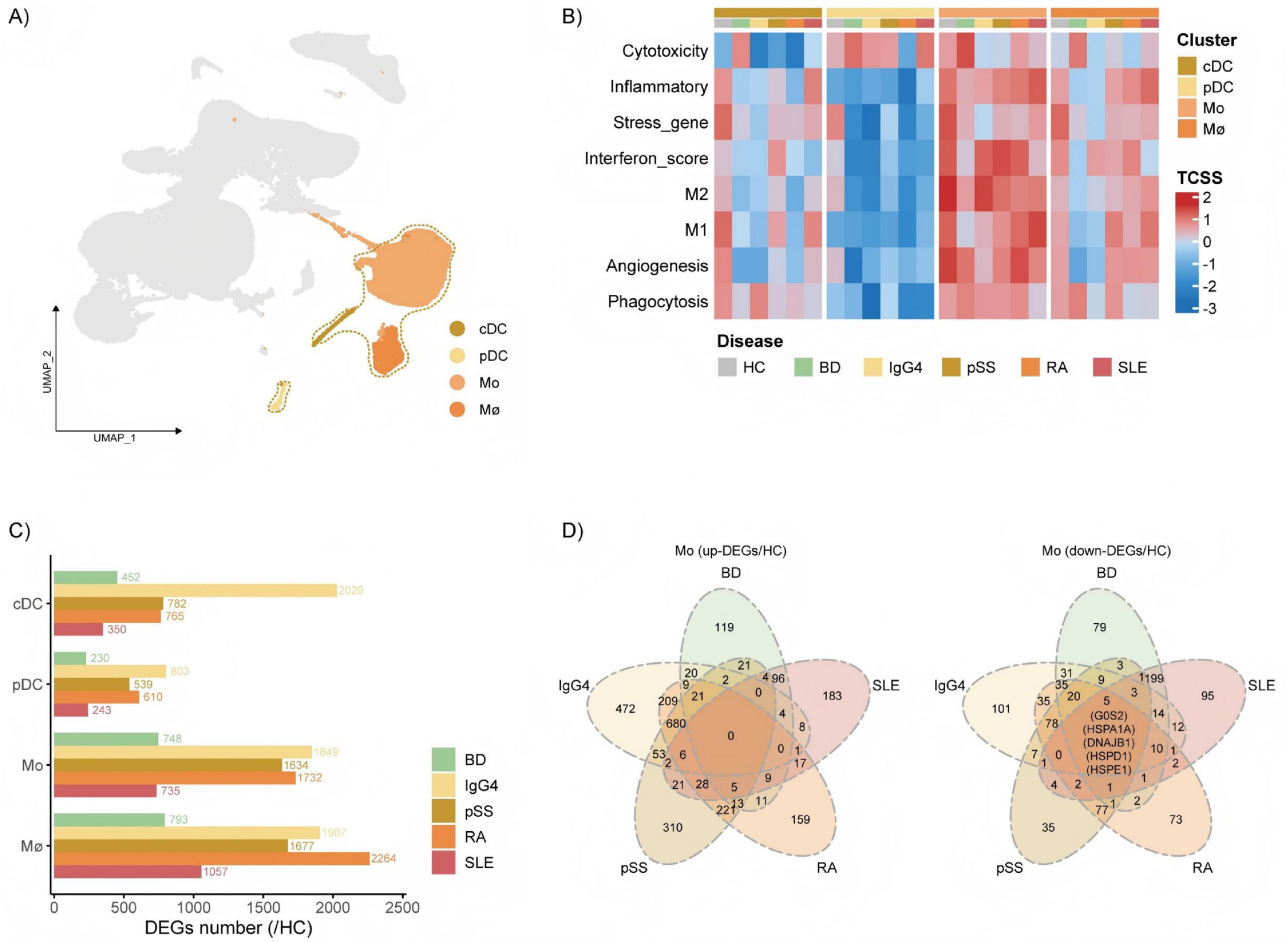
Differences of myeloid cells across rheumatic diseases. **(A)** Uniform manifold approximation and projection (UMAP) visualization of myeloid cells from all samples. Myeloid cell clusters are highlighted by dashed circles. **(B)** Heatmap of immune regulation-related functional signatures across myeloid cells. Scale the data in different T cell status score (TCSS) of all sub-clusters. **(C)** Barplots showing disease-specific differential genes expressions (DEGs) number for pDCs, cDCs, Mo, and Mø among the five diseases. **(D)** Venn plots showing the number of the same and the different disease-specific DEGs in rheumatic diseases Mo.

To further explore the heterogeneity of myeloid cells across rheumatic diseases, We evaluated the immune regulation-related characteristics of pDCs, cDCs, Mo, and Mø (Figures 3A, B). Monocytes showed a higher level of inflammatory response and interferon score (Supplementary Figure 3). Compared with other myeloid cells, the cytotoxicity, immune response and interferon scores of Mo were significantly increased across diseases, indicating that Mo have the highest immunomodulatory activity among peripheral blood myeloid cells. Among the myeloid cells, including cDCs, pDCs, Mo, and Mø both have relatively high cytotoxicity, and Mo and Mø also have highly active phagocytosis functions in the BD group. The inflammatory response activity of Mo and Mø is significantly higher in SLE than that of HC. In the Mø of SLE and RA, the M2 characteristics are significantly increased compared with HC, and the M2 activity is also higher than the M1 activity. Mo and Mø has more number of disease-specific DEGs than in any of the other sub-clusters (Figure 3C). The similarities between the up-regulated or down-regulated disease-specific DEGs across different diseases in each myeloid sub-clusters (Figure 3D and Supplementary Figure 3). We observed that the levels of heat shock proteins significantly decreased in macrophages of all the diseases. In addition, lgG4-RDs, RA, and pSS showed a high degree of similarity in disease-specific DEGs.

### 3.4 Interactions of major inflammatory cell types across diverse rheumatic diseases

**FIGURE 4:**
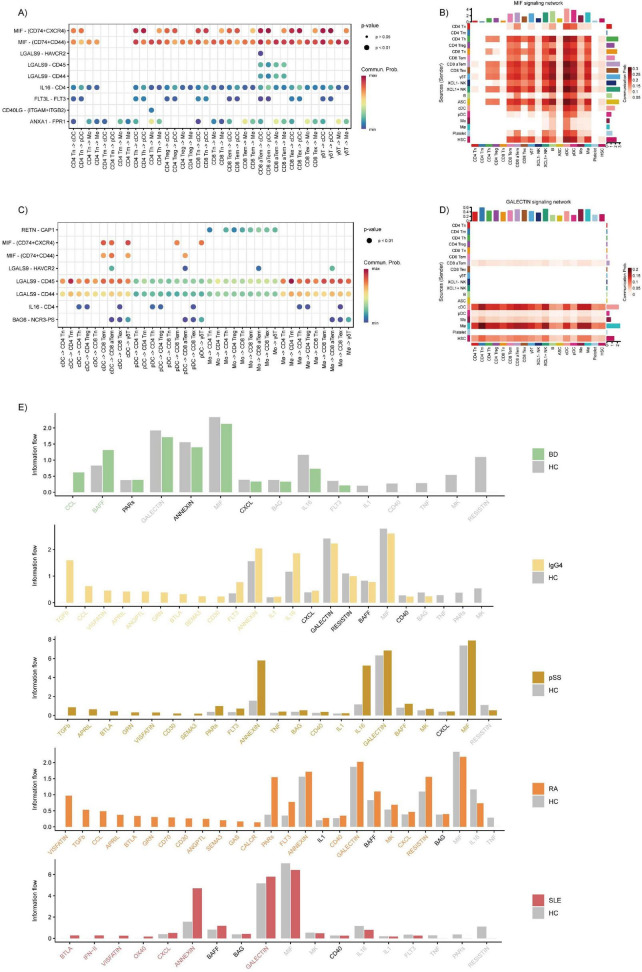
Cell interactions across diverse rheumatic diseases. **(A)** Dot plot showing the interactions from T cell sub-clusters to myeloid cells (pDCs, cDCs, Mo, Mø). **(B)** Heatmap of selected molecules showing the MIF signaling pathways for the cell types. **(C)** Dot plot showing the interactions from myeloid cells (pDCs, cDCs, Mo, Mø) to T cell sub-clusters. **(D)** Heatmap of selected molecules showing the GALECTIN signaling pathways for the cell types. **(E)** Barplots showing the different signaling pathways for all cell types among the five diseases.

To reveal the different characteristics of cell-to-cell communication in rheumatic diseases, we employed CellChat to infer the signaling network and ligand–receptor interactions among the major inflammatory cell types. We found that the MIF and GALECTIN signaling network were increased between T cell sub-clusters and myeloid cell clusters. Among them, T cell sub-clusters may regulate the migration of myeloid cells through MIF-related ligands (MIF-CD74+CD44/CXCR4) (Figures 4A, B), while myeloid cells, especially cDCs and macrophages, may regulate the immune function of T cells through the GALECTIN signaling pathway (LGALS9-CD44/CD45) (Figures 4C, D). Across rheumatic diseases, the MIF signaling network was decreased in four rheumatic diseases except pSS, compared with HC (Figure 4E). While the GALECTIN signaling network was decreased in BD and IgG4-RD compared with HC. Meanwhile, the MIF and GALECTIN signaling pathway shows significant differences between T cell sub-clusters and myeloid cell clusters across diverse rheumatic diseases (Supplementary Figures 4–8). The interaction intensity between PBMCs of pSS and RA is higher than that of HC, while it is lower in other diseases. Among all the diseases, the interaction between T cell subsets and myeloid cells (pDCs, cDCs, Mo, Mø) mainly focuses on the enhancement and weakening of different receptor-ligands in the MIF and GALECTIN signaling pathways. Moreover, we found that the activities of the MIF and GALECTIN signaling pathways are different in different diseases compared with HC, which may be related to the strength of the related receptor-ligands. Interestingly, we found that the receptor-ligands related to the MIF and GALECTIN signaling pathways in pSS and RA mainly increase in the diseases, which is different from the distribution of the receptor-ligands of the same signaling pathway in other diseases, which are evenly distributed in both diseases and normal conditions.

## 4 Discussion

This study integrated the single-cell transcriptome data of PBMC from five common rheumatic diseases to construct a cross-disease map containing over 246,000 immune cells. It systematically revealed the similarities and differences in the composition, functional status, gene expression, and cell-to-cell communication of immune cells among different rheumatic diseases.

Firstly, at the cellular composition level, we observed significant imbalances in the proportions of immune cells among different diseases. For instance, CD4^+^ T cells were significantly decreased in BD and RA, while CD8^+^ T cells were significantly decreased in IgG4-RD. The cell apoptosis and senescence of CD4+ T cells in BD lead to pro-inflammatory cytokines overproduction. Therefore, targeting CD4+ T cells might be a promising therapeutic strategy for BD (16). Similarly, multiple factors can promote the apoptosis of CD4+ T cells and the increase of cytotoxic responses, thereby causing inflammation in the RA tissues (17–19). Particularly noteworthy is that γδ T cells were consistently decreased in RA and SLE, suggesting that these innate-like T cells may be generally impaired in the immune regulation of some rheumatic diseases (20). γδ T cells exert significant influence on the progression, symptoms, and outcomes of rheumatoid arthritis by regulating the activity of αβ T cells and B cells that respond to classical self-antigens (21). Existing studies have enhanced the therapeutic potential of γδ T cells in rheumatic diseases through engineering methods such as CAR-γδ T cell therapy (20). In terms of T-cell function, we used the TCellSI tool to score the T cell status, and found that in the healthy control group, most T cells were in a resting state. Differential genes expression analysis further indicated that IgG4-RD and RA had more disease-specific DEGs in most T cell subsets, and both showed upregulation of LGALS1 and S100A4 in Treg cells, suggesting that these two diseases may have similar molecular changes in T cell regulation. Previous studies have found that Th17/Treg imbalance and dysregulation exist in the peripheral blood of IgG4-RD and RA, and are associated with the occurrence and progression of the diseases (22, 23). Additionally, heat shock proteins were generally down-regulated in CD8+ naive and memory T cells, suggesting that the cellular stress response pathway might be generally impaired in rheumatic disease T cells. During cellular stress, the concentration of the protein HSP60 encoded by HSPD1 increases. Hsp60 plays a role in rheumatoid arthritis by promoting the secretion of regulatory anti-inflammatory cytokines (such as IL-4 and IL-10) (24). Studies have found that inhibiting HSP60 can treat inflammatory responses in inflammatory diseases such as RA (25).

In the myeloid cells, macrophages were significantly increased in BD and RA, and monocytes and cDCs were also elevated in BD, indicating that the widespread activation of myeloid cells may be particularly associated with the inflammatory state of BD (15). The analysis of myeloid cells highlighted the functional differences between diseases. Monocytes and macrophages exhibited stronger phagocytic and cytotoxic functions in BD, while in SLE, they showed higher inflammatory response activity. It is notable that macrophages in SLE and RA both showed enhanced M2-type characteristics, suggesting that the polarization of anti-inflammatory/repairing macrophages may be commonly induced in chronic inflammatory environments. These results indicate that although the clinical manifestations of different rheumatic diseases vary, their immune cell infiltration patterns have certain overlaps, and also reflect the disease-specific immune deviations. Significant activation of the myeloid cell interferon pathway was not observed in SLE. This might be because we mainly focused on the overall changes of myeloid cells, and the confounding factors in the healthy control datasets might have affected the observation of some features. However, in the analysis of cell interactions, we did observe the activation of type II IFN related ligand-receptor pairs.

The CellChat analysis revealed the central role of the MIF and GALECTIN signaling pathways in the interaction between T cells and myeloid cells. Compared with healthy controls, the MIF signaling network was weakened in all four diseases except pSS, while the GALECTIN signal decreased in BD and IgG4-RD. MIF-dependent T cells may be associated with chronic inflammation and disease recurrence (26). The molecules in the GALECTIN signaling pathways, especially Galectin-9, can inhibit the promoting effect of MIF on chronic inflammation (27). Interestingly, the ligand-receptor interactions related to these two pathways were significantly enhanced in pSS and RA, and the distribution patterns were different from those in other diseases, suggesting that these two diseases may have unique immune regulatory characteristics at the cellular communication level. This finding provides a new perspective for further understanding the specific regulatory specificity of the immune network in different rheumatic diseases. Furthermore, the differences in the regulation of MIF and GALECTIN signaling pathways between T cells and myeloid cells in promoting and inhibiting inflammation reflect the balancing relationship of immune regulation. Future studies should experimentally validate the functional role of the MIF-GALECTIN axis using in vitro co-culture systems of T cells and macrophages from RA and pSS patients (28). Furthermore, the therapeutic potential of targeting γδ T function or Th17/Treg balance could be explored in murine models of IgG4-RD and RA (29).

This study also has certain limitations. All the data were derived from peripheral blood, failing to reflect the changes in the local immune microenvironment of tissues; the sample size was not balanced among different diseases, which may affect the statistical power; in addition, the scRNA-seq data did not provide protein-level or functional experimental verification. Future research can combine multi-tissue sampling, spatial transcriptomics, and *in vitro* functional experiments to further validate and expand the pathways and mechanisms discovered in this study. Our cross-disease analysis aggregates single-cell data from multiple donors, which inherently averages out inter-individual genetic variability to reveal shared disease-associated signals. However, this approach cannot capture the impact of specific host genetic variants on cellular phenotypes. Future studies integrating scRNA-seq with donor genotyping could address this question.

In summary, through the cross-analysis of single-cell transcriptomes across various rheumatic diseases, this study revealed the shared immune cell dysregulation patterns (such as decreased γδ T cells and downregulated heat shock proteins) among different diseases, as well as disease-specific immune characteristics (such as differences in activation of cell communication pathways). These findings not only deepen our understanding of the common and individual immune pathologies in rheumatic diseases, but also provide an important basis for developing immunotherapy strategies targeting shared pathways or disease-specific mechanisms.

## Data Availability

The original contributions presented in this study are included in the article/Supplementary material, further inquiries can be directed to the corresponding author.
