# Supplementary material for: Cross-disease immune cells atlas reveals the similarities and differences of cell characteristics and interactions in rheumatic diseases
Source: Front Med (Lausanne). 2026 May 13;13:1820336. doi: 10.3389/fmed.2026.1820336 (PMC13212184; doi:10.3389/fmed.2026.1820336)
Supplement: Supplementary file 1 [file Data_Sheet_1.pdf]

Table S1. Summary of the public scRNA-seq datasets used in this study.

Dataset ID	Disease	Number of Patients	Number of HC	Patient Status	scRNA-seq Platform	Data Accession	Key Reference (PMID)
GSE198616	BD	4	4	Active, untreated	10X Genomics	GEO	PMID: 35727985
GSE157278	pSS	5	5	Active, untreated	10X Genomics	GEO	PMID: 33603736
GSE135779	SLE	7	5	Active, untreated	10X Genomics	GEO	PMID: 32747814
GSE159117	RA	1	0	Active, untreated	10X Genomics	GEO	PMID: 35015026
HRA000916	RA	1	1	Active, untreated	10X Genomics	Genome Sequence Archive for Human	PMID: 34462261
HRA001555	IgG4-RD	4	3	Active, untreated	10X Genomics	Genome Sequence Archive for Human	PMID: 35693817

HC (healthy controls), RA (rheumatoid arthritis), SLE (systemic lupus erythematosus), pSS (primary Sjögren's syndrome), BD (Behçet's disease), and IgG4-RD (IgG4-related disease).

Table S2: immune regulation-related functional signatures

Gene	signatures
GZMA	cytotoxicity
GZMB	cytotoxicity
GZMH	cytotoxicity
GZMM	cytotoxicity
GZMK	cytotoxicity
GNLY	cytotoxicity
PRF1	cytotoxicity
CTSW	cytotoxicity
CCL5	inflammatory
CXCL10	inflammatory
CXCL9	inflammatory
IL1B	inflammatory
IL6	inflammatory
IL7	inflammatory
IL15	inflammatory
IL18	inflammatory
CCL2	inflammatory
CCL3	inflammatory
CCL4	inflammatory
BAG3	stress_gene
CALU	stress_gene
DNAJB1	stress_gene
DUSP1	stress_gene

EGR1	stress_gene
FOS	stress_gene
FOSB	stress_gene
HIF1A	stress_gene
HSP90AA1	stress_gene
HSP90AB1	stress_gene
HSP90B1	stress_gene
HSPA1A	stress_gene
HSPA1B	stress_gene
HSPA6	stress_gene
HSPB1	stress_gene
HSPH1	stress_gene
IER2	stress_gene
JUN	stress_gene
JUNB	stress_gene
NFKBIA	stress_gene
NFKBIZ	stress_gene
RGS2	stress_gene
SLC2A3	stress_gene
SOCS3	stress_gene
UBC	stress_gene
ZFAND2A	stress_gene
ZFP36	stress_gene
ZFP36L1	stress_gene
AAAS	Interferon_score
ABCE1	Interferon_score
ADAR	Interferon_score
ARIH1	Interferon_score
B2M	Interferon_score
BECN1	Interferon_score
BST2	Interferon_score
CAMK2A	Interferon_score
CAMK2B	Interferon_score
CAMK2D	Interferon_score
CAMK2G	Interferon_score
CD44	Interferon_score
CDK1	Interferon_score
CENPS	Interferon_score
CENPX	Interferon_score
CHUK	Interferon_score
CIITA	Interferon_score
DHX9	Interferon_score
DNAJC3	Interferon_score
DUS2	Interferon_score

EGR1	Interferon_score
EIF2AK2	Interferon_score
EIF2S1	Interferon_score
EIF2S2	Interferon_score
EIF2S3	Interferon_score
EIF4A1	Interferon_score
EIF4A2	Interferon_score
EIF4A3	Interferon_score
EIF4E	Interferon_score
EIF4E2	Interferon_score
EIF4E3	Interferon_score
EIF4G1	Interferon_score
EIF4G2	Interferon_score
EIF4G3	Interferon_score
FAAP100	Interferon_score
FAAP20	Interferon_score
FAAP24	Interferon_score
FANCA	Interferon_score
FANCB	Interferon_score
FANCC	Interferon_score
FANCE	Interferon_score
FANCF	Interferon_score
FANCG	Interferon_score
FANCL	Interferon_score
FANCM	Interferon_score
FCGR1A	Interferon_score
FCGR1BP	Interferon_score
FLNA	Interferon_score
FLNB	Interferon_score
GBP1	Interferon_score
GBP2	Interferon_score
GBP3	Interferon_score
GBP4	Interferon_score
GBP5	Interferon_score
GBP6	Interferon_score
GBP7	Interferon_score
HERC5	Interferon_score
HLA-A	Interferon_score
HLA-B	Interferon_score
HLA-C	Interferon_score
HLA-DPA1	Interferon_score
HLA-DPB1	Interferon_score
HLA-DQA1	Interferon_score
HLA-DQA2	Interferon_score

HLA-DQB1	Interferon_score
HLA-DQB2	Interferon_score
HLA-DRA	Interferon_score
HLA-DRB1	Interferon_score
HLA-DRB3	Interferon_score
HLA-DRB4	Interferon_score
HLA-DRB5	Interferon_score
HLA-E	Interferon_score
HLA-F	Interferon_score
HLA-G	Interferon_score
HLA-H	Interferon_score
HSPA1A	Interferon_score
HSPA1B	Interferon_score
HSPA1L	Interferon_score
HSPA2	Interferon_score
HSPA8	Interferon_score
ICAM1	Interferon_score
IFI27	Interferon_score
IFI30	Interferon_score
IFI35	Interferon_score
IFI6	Interferon_score
IFIT1	Interferon_score
IFIT2	Interferon_score
IFIT3	Interferon_score
IFIT5	Interferon_score
IFITM1	Interferon_score
IFITM2	Interferon_score
IFITM3	Interferon_score
IFNA1	Interferon_score
IFNA10	Interferon_score
IFNA13	Interferon_score
IFNA14	Interferon_score
IFNA16	Interferon_score
IFNA17	Interferon_score
IFNA2	Interferon_score
IFNA21	Interferon_score
IFNA4	Interferon_score
IFNA5	Interferon_score
IFNA6	Interferon_score
IFNA7	Interferon_score
IFNA8	Interferon_score
IFNAR1	Interferon_score
IFNAR2	Interferon_score
IFNB1	Interferon_score

IFNG	Interferon_score
IFNGR1	Interferon_score
IFNGR2	Interferon_score
IKBKB	Interferon_score
IKBKG	Interferon_score
ILF2	Interferon_score
ILF3	Interferon_score
IP6K2	Interferon_score
IRF1	Interferon_score
IRF2	Interferon_score
IRF3	Interferon_score
IRF4	Interferon_score
IRF5	Interferon_score
IRF6	Interferon_score
IRF7	Interferon_score
IRF8	Interferon_score
IRF9	Interferon_score
ISG15	Interferon_score
ISG20	Interferon_score
JAK1	Interferon_score
JAK2	Interferon_score
KPNA1	Interferon_score
KPNA2	Interferon_score
KPNA3	Interferon_score
KPNA4	Interferon_score
KPNA5	Interferon_score
KPNA7	Interferon_score
KPNB1	Interferon_score
MAP2K6	Interferon_score
MAPK1	Interferon_score
MAPK3	Interferon_score
MAPT	Interferon_score
MAVS	Interferon_score
MID1	Interferon_score
MT2A	Interferon_score
MX1	Interferon_score
MX2	Interferon_score
NCAM1	Interferon_score
NCK1	Interferon_score
NDC1	Interferon_score
NEDD4	Interferon_score
NPM1	Interferon_score
NUP107	Interferon_score
NUP133	Interferon_score

NUP153	Interferon_score
NUP155	Interferon_score
NUP160	Interferon_score
NUP188	Interferon_score
NUP205	Interferon_score
NUP210	Interferon_score
NUP214	Interferon_score
NUP35	Interferon_score
NUP37	Interferon_score
NUP42	Interferon_score
NUP43	Interferon_score
NUP50	Interferon_score
NUP54	Interferon_score
NUP58	Interferon_score
NUP62	Interferon_score
NUP85	Interferon_score
NUP88	Interferon_score
NUP93	Interferon_score
NUP98	Interferon_score
OAS1	Interferon_score
OAS2	Interferon_score
OAS3	Interferon_score
OASL	Interferon_score
PDE12	Interferon_score
PIAS1	Interferon_score
PIN1	Interferon_score
PLCG1	Interferon_score
PML	Interferon_score
POM121	Interferon_score
POM121C	Interferon_score
PPM1B	Interferon_score
PPP2CA	Interferon_score
PPP2CB	Interferon_score
PPP2R1A	Interferon_score
PPP2R1B	Interferon_score
PPP2R5A	Interferon_score
PRKCD	Interferon_score
PRKRA	Interferon_score
PSMB8	Interferon_score
PTAFR	Interferon_score
PTPN1	Interferon_score
PTPN11	Interferon_score
PTPN2	Interferon_score
PTPN6	Interferon_score

RAE1	Interferon_score
RAF1	Interferon_score
RANBP2	Interferon_score
RIGI	Interferon_score
RNASEL	Interferon_score
RPS27A	Interferon_score
RSAD2	Interferon_score
SAMHD1	Interferon_score
SEC13	Interferon_score
SEH1L	Interferon_score
SMAD7	Interferon_score
SNCA	Interferon_score
SOCS1	Interferon_score
SOCS3	Interferon_score
SP100	Interferon_score
SPHK1	Interferon_score
STAT1	Interferon_score
STAT2	Interferon_score
STAT3	Interferon_score
SUMO1	Interferon_score
TARBP2	Interferon_score
TP53	Interferon_score
TPR	Interferon_score
TRIM10	Interferon_score
TRIM14	Interferon_score
TRIM17	Interferon_score
TRIM2	Interferon_score
TRIM21	Interferon_score
TRIM22	Interferon_score
TRIM25	Interferon_score
TRIM26	Interferon_score
TRIM29	Interferon_score
TRIM3	Interferon_score
TRIM31	Interferon_score
TRIM34	Interferon_score
TRIM35	Interferon_score
TRIM38	Interferon_score
TRIM45	Interferon_score
TRIM46	Interferon_score
TRIM48	Interferon_score
TRIM5	Interferon_score
TRIM6	Interferon_score
TRIM62	Interferon_score
TRIM68	Interferon_score

TRIM8	Interferon_score
TUBA1A	Interferon_score
TUBA1B	Interferon_score
TUBA1C	Interferon_score
TUBA3C	Interferon_score
TUBA3D	Interferon_score
TUBA3E	Interferon_score
TUBA4A	Interferon_score
TUBA4B	Interferon_score
TUBA8	Interferon_score
TUBAL3	Interferon_score
TUBB1	Interferon_score
TUBB2A	Interferon_score
TUBB2B	Interferon_score
TUBB3	Interferon_score
TUBB4A	Interferon_score
TUBB4B	Interferon_score
TUBB6	Interferon_score
TUBB8	Interferon_score
TUBB8B	Interferon_score
TYK2	Interferon_score
UBA52	Interferon_score
UBA7	Interferon_score
UBB	Interferon_score
UBC	Interferon_score
UBE2E1	Interferon_score
UBE2I	Interferon_score
UBE2L6	Interferon_score
UBE2N	Interferon_score
USP18	Interferon_score
USP41	Interferon_score
VCAM1	Interferon_score
XAF1	Interferon_score
YBX1	Interferon_score
IL4R	M2
CCL4	M2
CCL13	M2
CCL20	M2
CCL17	M2
CCL18	M2
CCL22	M2
CCL24	M2
LYVE1	M2
VEGFA	M2

VEGFB	M2
VEGFC	M2
VEGFD	M2
EGF	M2
CTSA	M2
CTSB	M2
CTSC	M2
CTSD	M2
TGFB1	M2
TGFB2	M2
TGFB3	M2
MMP14	M2
MMP19	M2
MMP9	M2
CLEC7A	M2
WNT7B	M2
FASL	M2
TNFSF12	M2
TNFSF8	M2
CD276	M2
VTCN1	M2
MSR1	M2
FN1	M2
IRF4	M2
IL23	M1
TNF	M1
CXCL9	M1
CXCL10	M1
CXCL11	M1
CD86	M1
IL1A	M1
IL1B	M1
IL6	M1
CCL5	M1
IRF5	M1
IRF1	M1
CD40	M1
IDO1	M1
KYNU	M1
CCR7	M1
CCND2	Angiogenesis
CCNE1	Angiogenesis
CD44	Angiogenesis
CXCR4	Angiogenesis

E2F3	Angiogenesis
EDN1	Angiogenesis
EZH2	Angiogenesis
FGF18	Angiogenesis
FGFR1	Angiogenesis
FYN	Angiogenesis
HEY1	Angiogenesis
ITGAV	Angiogenesis
JAG1	Angiogenesis
JAG2	Angiogenesis
MMP9	Angiogenesis
NOTCH1	Angiogenesis
PDGFA	Angiogenesis
PTK2	Angiogenesis
SPP1	Angiogenesis
STC1	Angiogenesis
TNFAIP6	Angiogenesis
TYMP	Angiogenesis
VAV2	Angiogenesis
VCAN	Angiogenesis
VEGFA	Angiogenesis
MRC1	Phagocytosis
CD163	Phagocytosis
MERTK	Phagocytosis
C1QB	Phagocytosis
