# Supplementary material for: Cross-disease immune cells atlas reveals the similarities and differences of cell characteristics and interactions in rheumatic diseases
Source: Front Med (Lausanne). 2026 May 13;13:1820336. doi: 10.3389/fmed.2026.1820336 (PMC13212184; doi:10.3389/fmed.2026.1820336)
Supplement: Supplementary file 2 [file Data_Sheet_2.pdf]

SFigure 1

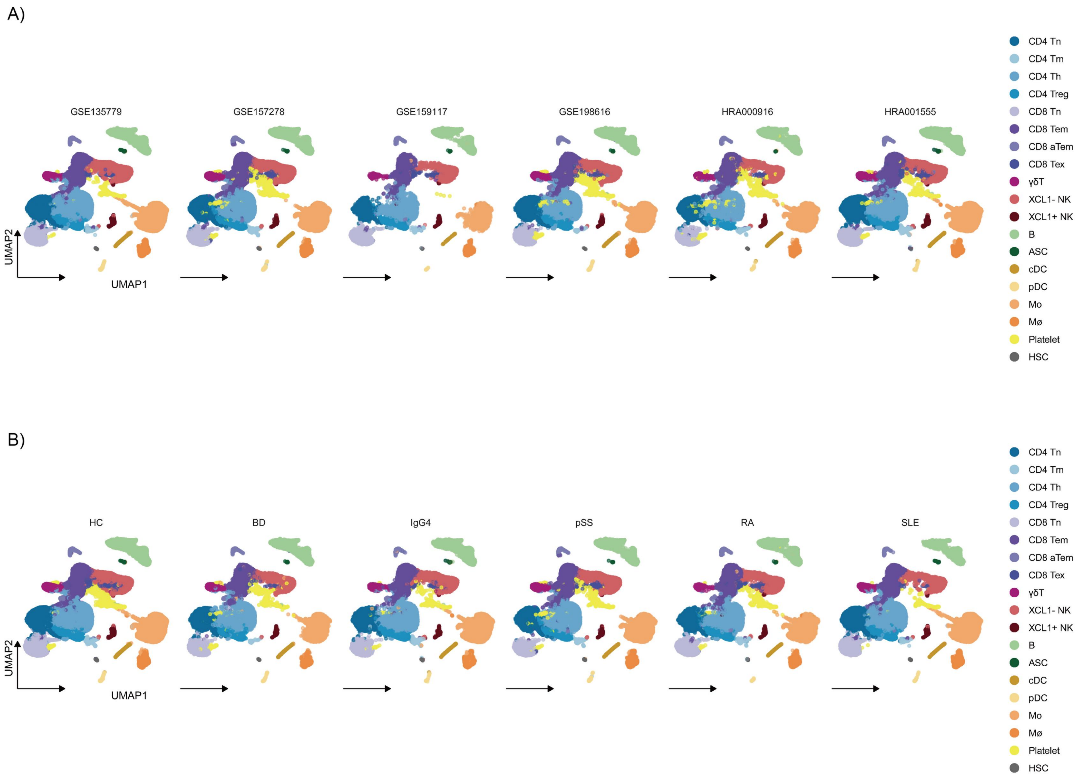

SFigure 1: scRNA-seq atlas of PBMCs across diverse, related to Figure 1. A) UMAP plots showing the cell compositions at a single sample level. B) cell compositions at the data sources level.

SFigure 2

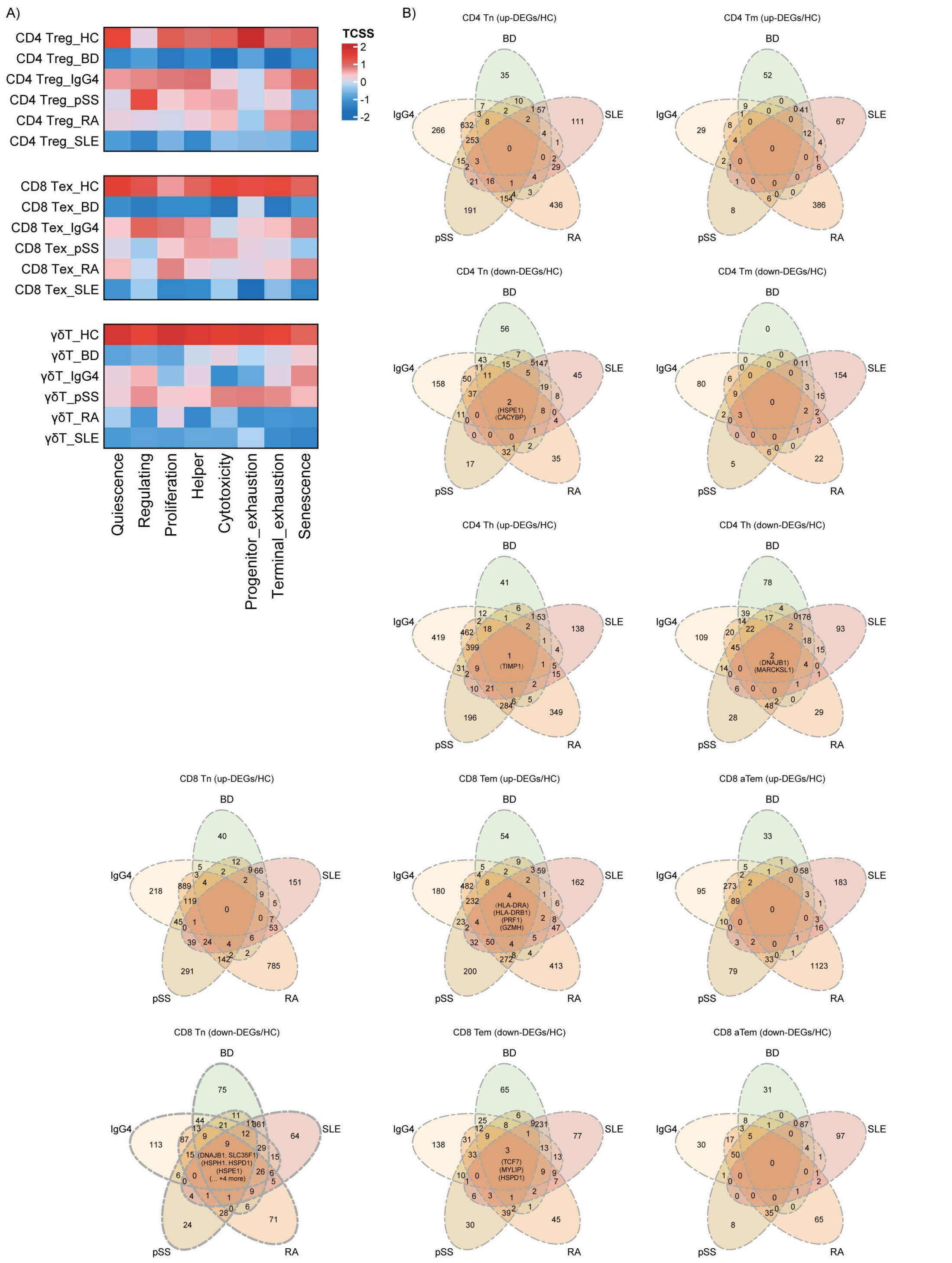

SFigure 2: Similar T cell dysfunction in rheumatic diseases, related to Figure 2. A) Heatmap of T cell status score (TCSS) across samples of a sub-clusters. B) Venn plots showing the number of the same and the different disease-specific DEGs in rheumatic diseases T cell sub-clusters.

SFigure 3

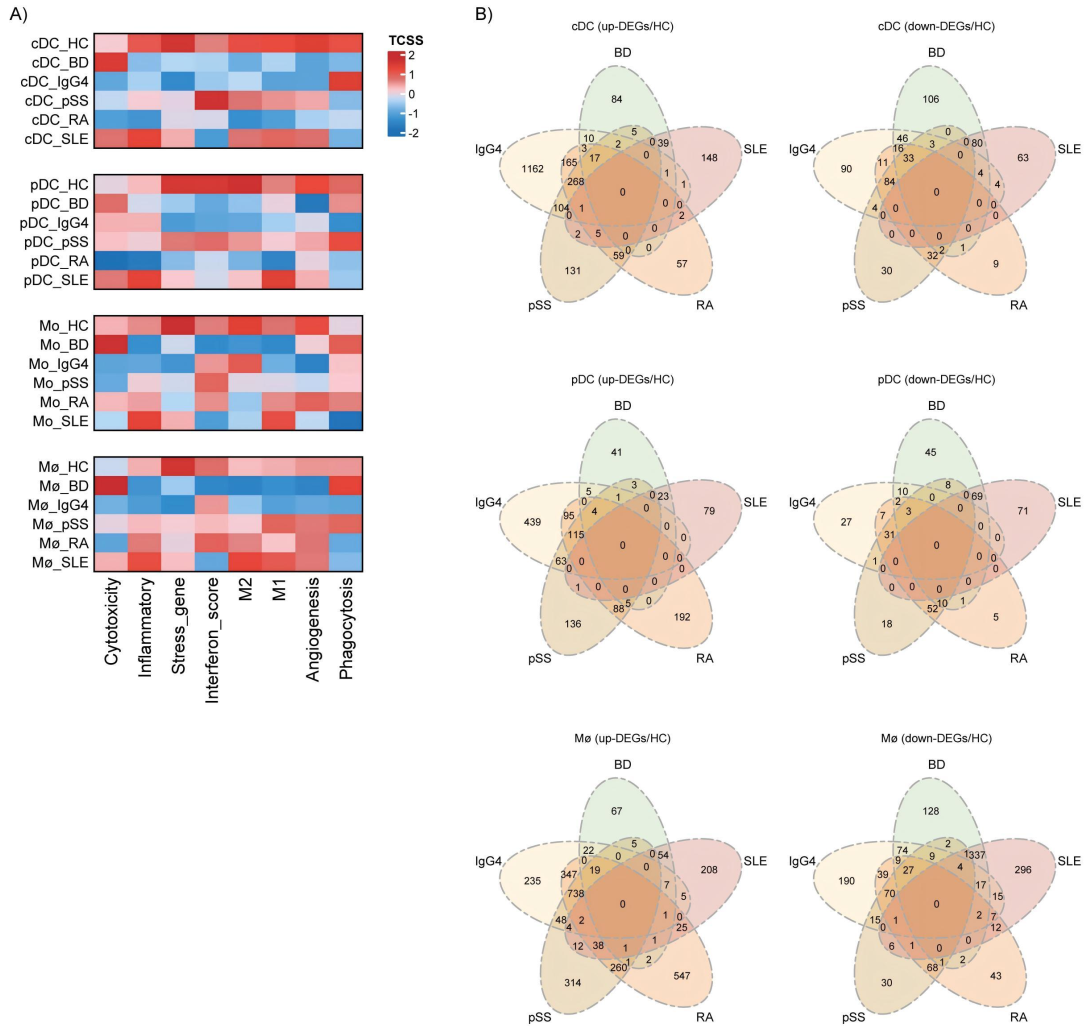

SFigure 3: Differences of myeloid cells across rheumatic diseases, related to Figure 3. A) Heatmap of immune regulation relatedt functional signatures across samples of a sub-cluster. B) Venn plots showing the number of the same and the different disease-specific DEGs in rheumatic diseases pDCs, cDCs, and Mø.

SFigure 4

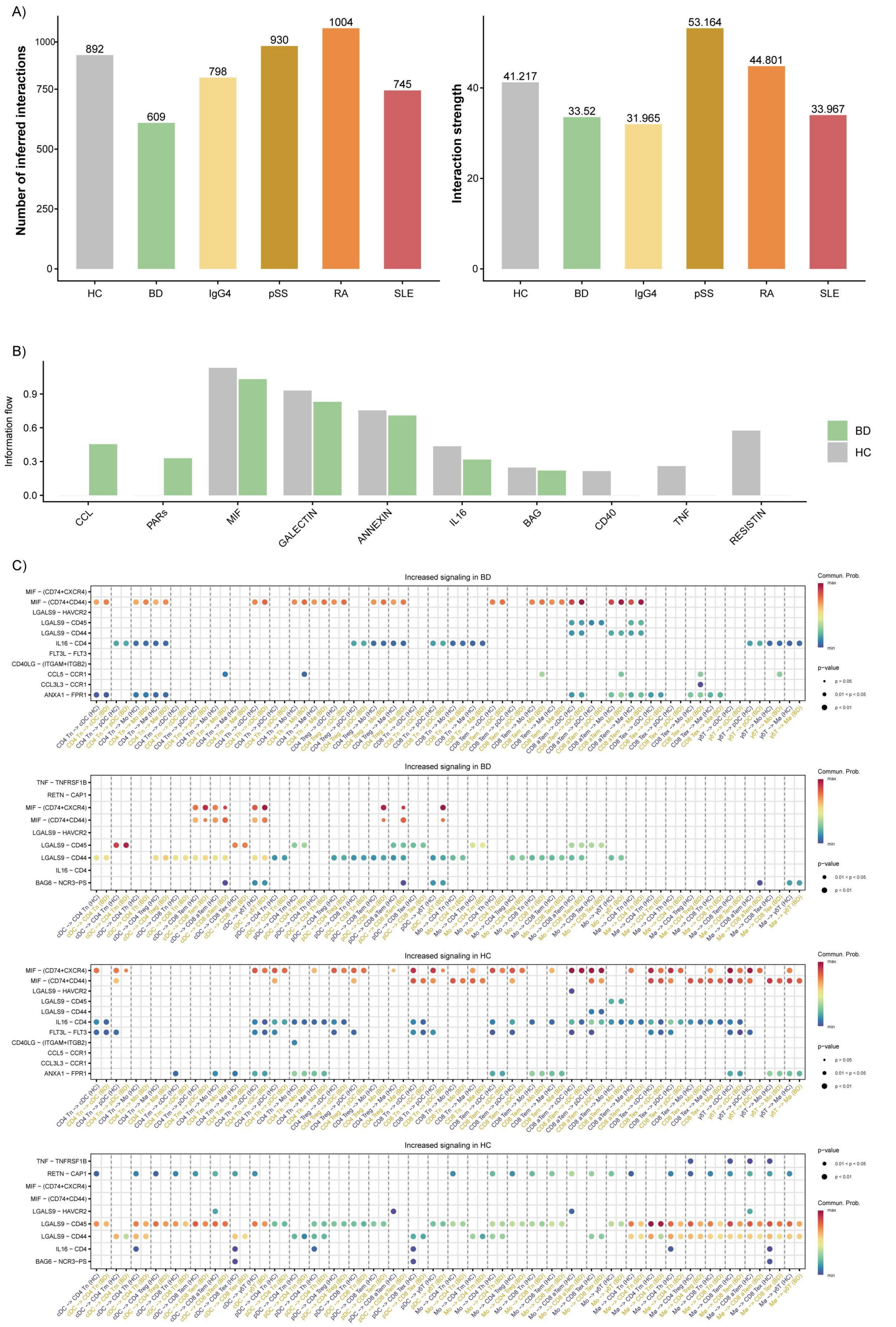

SFigure 4: Cell interactions across diverse rheumatic diseases, related to Figure 4. A) Quantity and intensity of the overall signaling among the five diseases. B) Barplots showing the different signaling pathways for T cell and myeloid cells in BD or HC. C) Dot plot showing the interactions between T cell sub-clusters and myeloid cells (pDCs, cDCs, Mo, Mø) in BD and HC.

SFigure 5

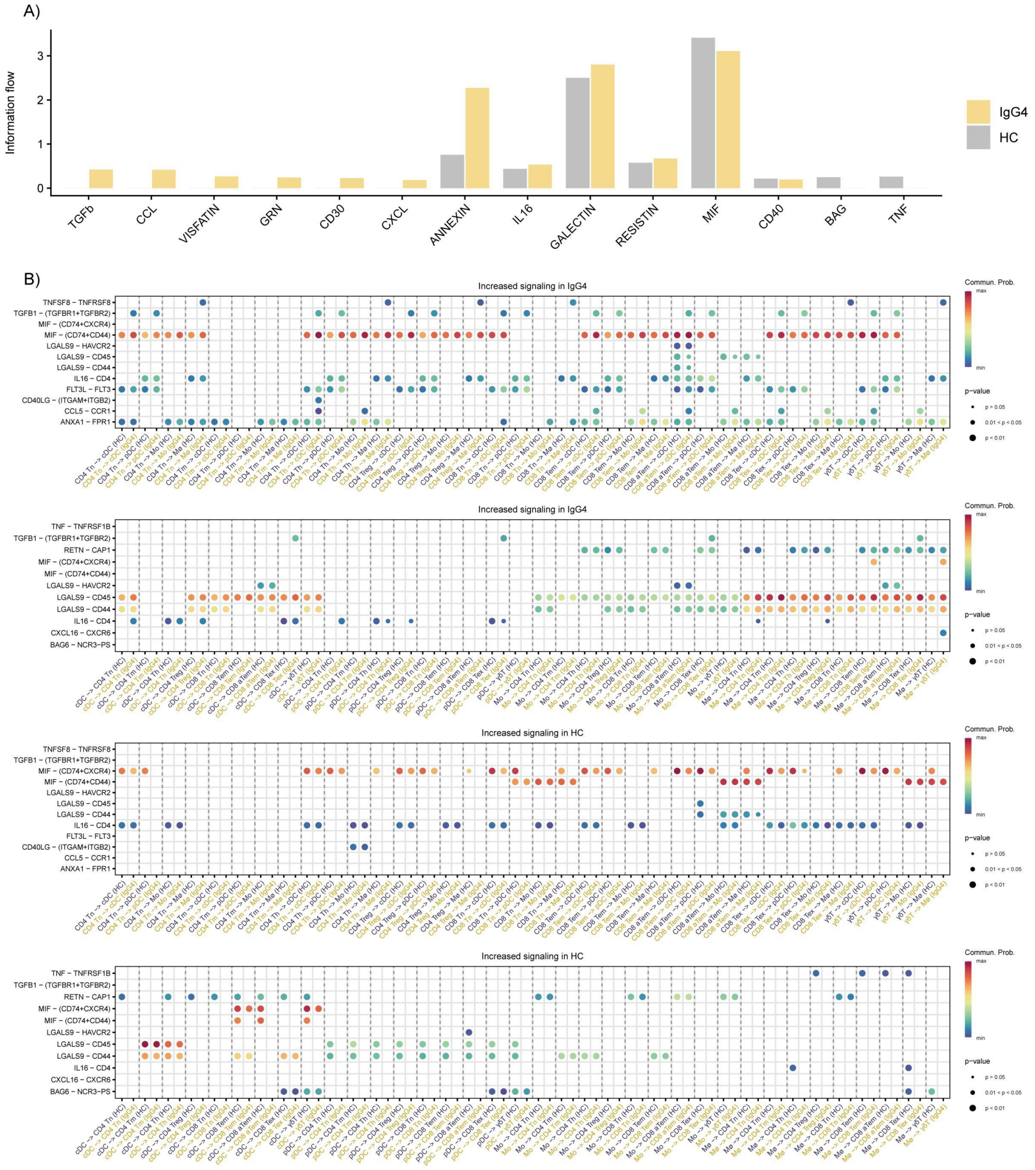

SFigure 5: Cell interactions in IgG4-RDs. A) Barplots showing the different signaling pathways for T cell and myeloid cells in IgG4-RDs or HC. B) Dot plot showing the interactions between T cell sub-clusters and myeloid cells (pDCs, cDCs, Mo, Mø) in IgG4-RDs and HC.

SFigure 6

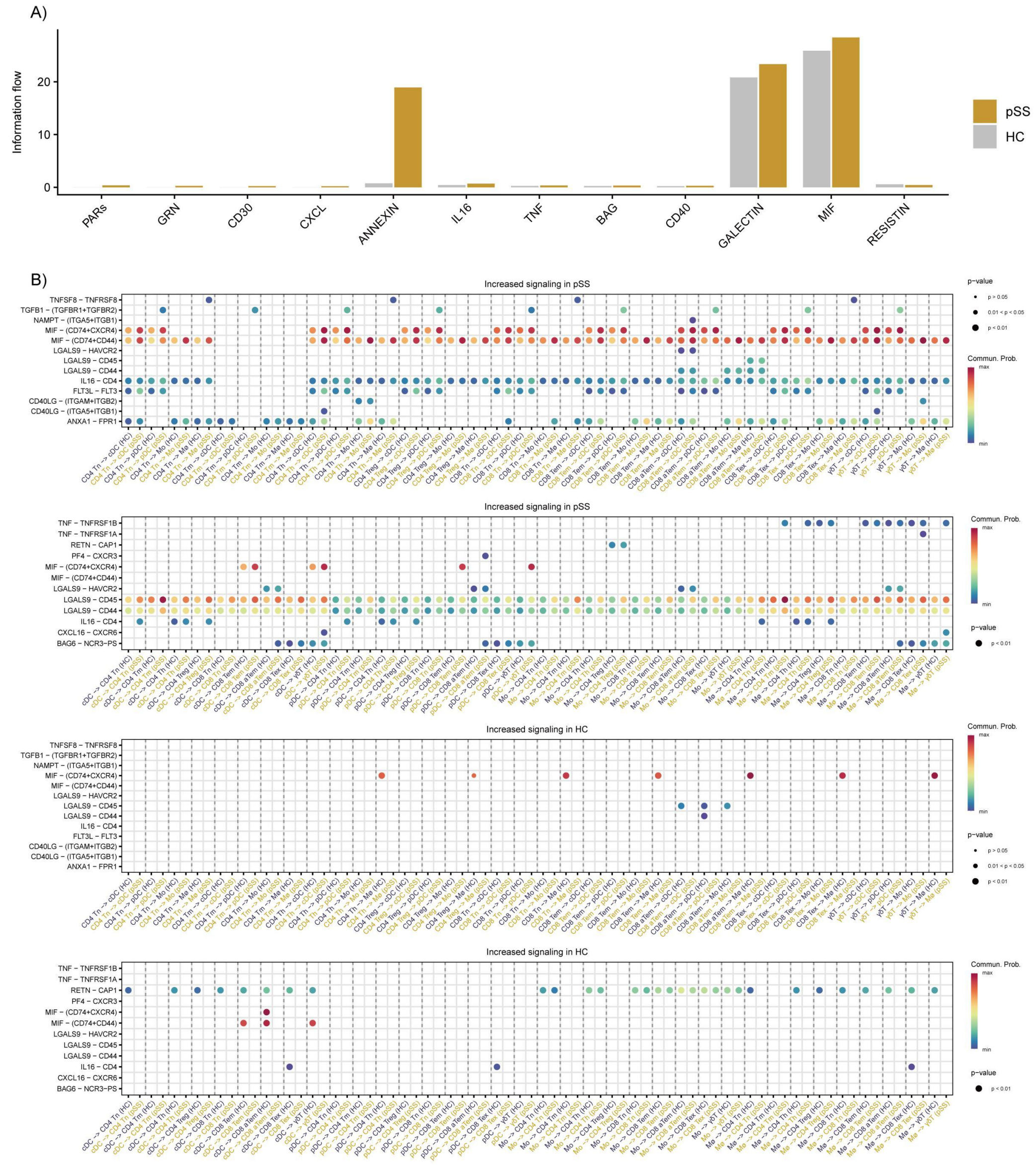

SFigure 6: Cell interactions in pSS. A) Barplots showing the different signaling pathways for T cell and myeloid cells in pSS or HC. B) Dot plot showing the interactions between T cell sub-clusters and myeloid cells (pDCs, cDCs, Mo, Mø) in pSS and HC.

SFigure 7

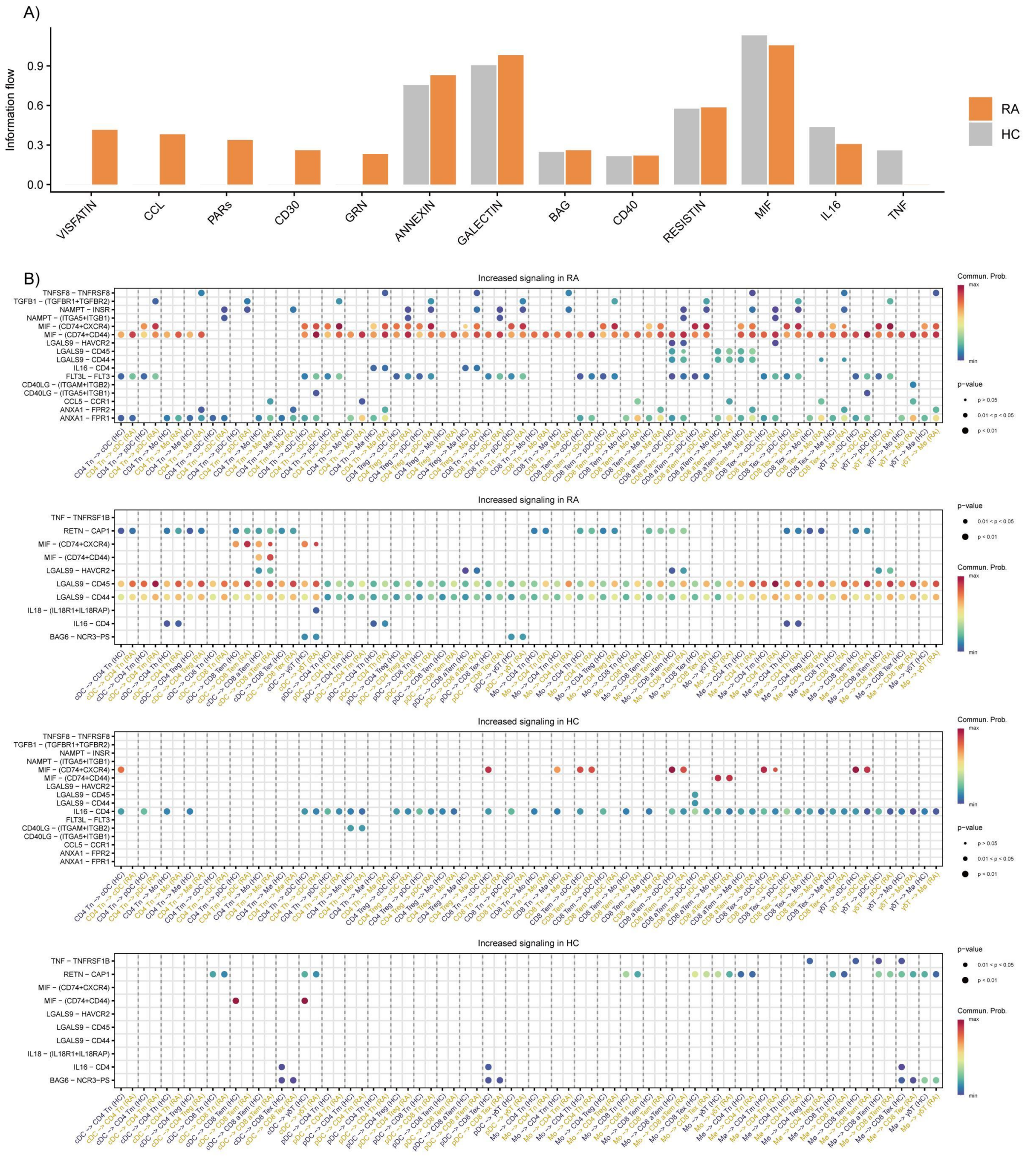

SFigure 7: Cell interactions in RA. A) Barplots showing the different signaling pathways for T cell and myeloid cells in RA or HC. B) Dot plot showing the interactions between T cell sub-clusters and myeloid cells (pDCs, cDCs, Mo, Mø) in RA and HC.

SFigure 8

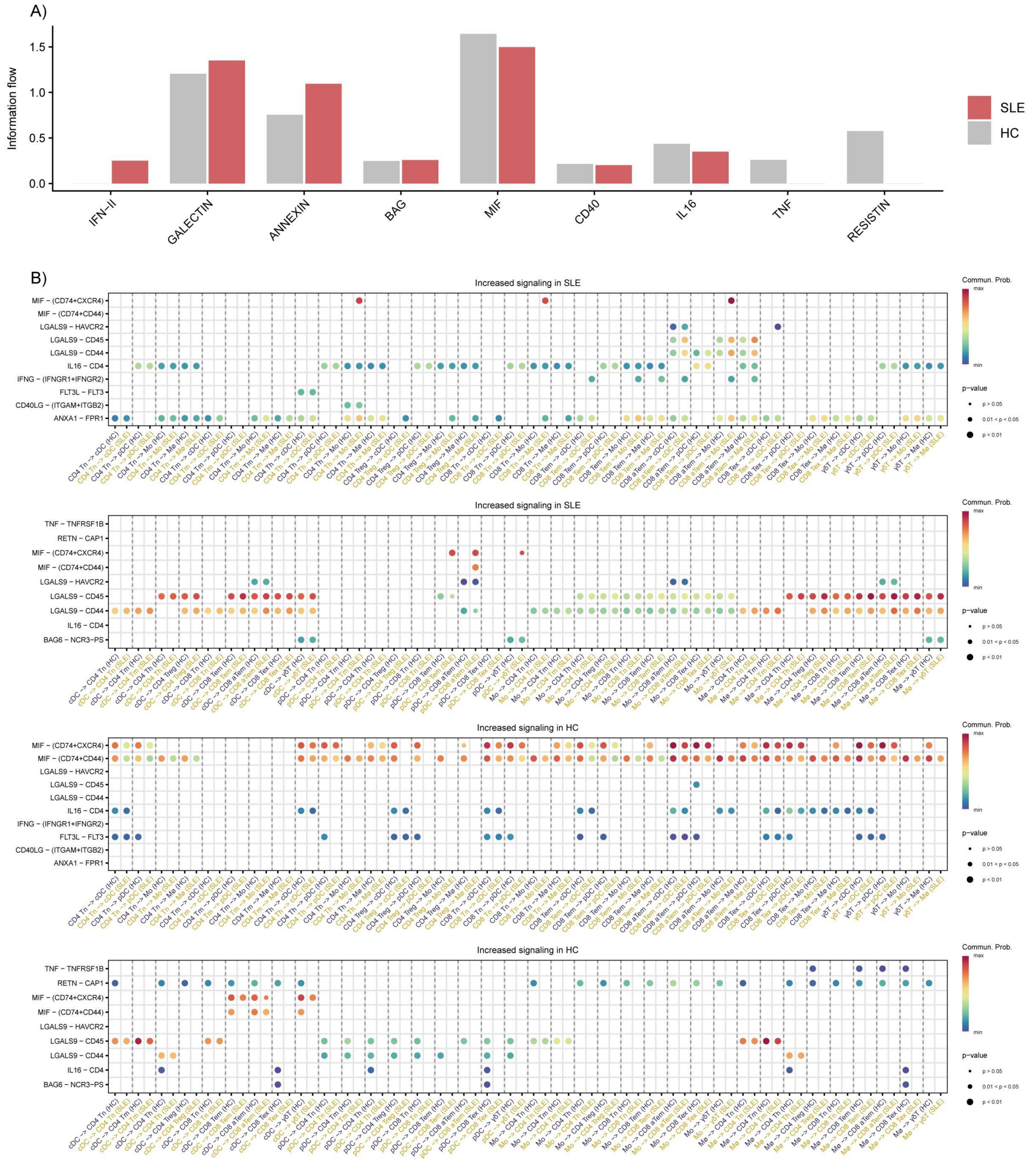

SFigure 8: Cell interactions in SLE. A) Barplots showing the different signaling pathways for T cell and myeloid cells in SLE or HC. B) Dot plot showing the interactions between T cell sub-clusters and myeloid cells (pDCs, cDCs, Mo, Mø) in SLE and HC.
